# 

**Published:** 1880-10

**Authors:** 


### HYMENEAL.

"Domestic happiness, thou only bliss
Of paradise that has survived the fall."

Married, August 26th, 1880, at Ellesslie Place, residence of the bride's parents, near Petersburg, Va., by the Rev. Mr. Logan,

Frank M. Earle, M. D., of Philadelphia, and Mary E. Vesey, formerly of Philadelphla.

Married, at Memorial P. E. Church, by Rev. Wm. M. Dame, Rector, on Wednesday evening, October the 6th, 1880, Silas W. Hunter, M. D., and Miss Sarah J. Henderson, daughter of Mrs. C. L. Henderson, all of this city.

We congratulate our friend and former pupil upon his auspicious entrance into the matrimonial ranks, and wish him and his charming bride a bright and prosperous future.

---

### NATALITIAL.

"Of all the joys that brighten suffering earth,
What joy is welcomed like a new-born child?"

Born at Whitesville, Ind., on the 15th of August, a daughter to Dr. and Mrs. Dudley M. Culver.

WILL *YOU* PLEASE SEND IN *YOUR* SUBSCRIPTION MONEY NOW?

Many persons who receive pamphlets, papers, etc., through the mails are unaware of the United States laws relating to them, and such will feel indebted to us for the information and guidance the enactments will afford which we copy below, viz:

1. A postmaster is required to give notice *by letter* (returning a paper does not answer the law) when a subscriber does not take his paper out of the office, and state the reason for its not being taken. Any neglect to do so makes the postmaster *responsible* to the publishers for payment.

2. Any person who takes a paper from the post-office, whether directed to his name or another, or whether he has subscribed or not, is responsible for the pay.

3. If a person orders his paper discontinued, he must pay all arrearges, or the publisher may continue to send it until payment is made, and collect the whole amount, *whether it be taken from the office or not*. There can be no legal discontinuance until the payment is made.

4. If the subscriber orders his paper to be stopped at a certain time, and the publisher continue to send, the subscriber is bound to pay for it *if he takes it out of the post-office*. The law proceeds upon the ground that a man must pay for what he uses.

5. The courts have decided that refusing to take newspaper and periodicals from the post-office or removing and leaving them uncalled for, is *prima facie* evidence of intentional fraud.

---

This column will be sold for advertising space six month during the year at *special rates*.

N B—Each month THE INDEPENDENT PRACTITIONER will be received by a large number of persons for the *first* time, all of whom we trust will be induced to become subscribers. They will please remit NOW the amount of subscription ($2.00) to insure the continuation of the journal. Money can be sent at our risk by *post-office money order*, registered letter or checks on banks in *N. Y. City or Baltimore, made payable to Dr. B. M. Wilkerson*. We hope that the few who have been receiving the journal and have not "paid up" will cheerfully do so *now*.

